# Study of Glove Perforation during Hip Replacement Arthroplasty: Its Frequency, Location, and Timing

**DOI:** 10.1155/2014/129561

**Published:** 2014-10-29

**Authors:** Li Xiao Tao, Deepak Kumar Basnet

**Affiliations:** Department of Orthopedics, The First Affiliated Hospital of Jiamusi University, Unit 3, 148 Xuefu Street, Xiangyang, Jiamusi, Heilongjiang 154007, China

## Abstract

*Objective*. The aim of the study was to evaluate the location, timing, and frequency of glove perforation during hip replacement arthroplasty. *Methods*. Gloves worn by surgical team members in 19 primary hip replacement arthroplasties were assessed. The study was of a single gloving system. All the used gloves were collected at the end of the surgery and assessed visually and by using water inflation technique. Relevant data were collected at the time of surgery. *Results*. A total of one hundred and ninety-one surgical gloves were evaluated. Twenty-three glove perforations were noted in nineteen of the operations. Of these perforations 14 belonged to gloves worn by surgeon and first assistant (60.1%). Glove perforation in thumb, index finger, and palm was more common. More perforation occurred in the gloves worn in nondominant hand (52%) but was insignificant. *Conclusion*. Glove perforation in surgeries such as total hip arthroplasty is not uncommon. In this study of single gloving system glove perforation rate was 12.04%, whereas literature reports of glove perforation rate as low as 3.3% in elective orthopedic surgeries with double gloving system. As such emphasis should be given to wear double pair of gloves wherever this practice is uncommon.

## 1. Introduction

Hip replacement arthroplasty is a major orthopedic surgery. Every surgery is an invasive procedure and with it comes the risks of exposure to blood and body fluids, like synovial fluids, saliva, urine, intestinal contents, and so forth, and risks of transfer of pathogens between the operating team and the patient. It is the surgical glove that offers protection against infection transmission between perioperative staffs and the patient [1]. The role of glove in reducing postoperative infections is now well understood. However, failure of glove during use can occur. Using double gloves and indicator gloves and changing gloves at regular intervals and before specific procedure during surgery are some of the methods that can be used to reduce risks involved while operating [2].

Surgery is the “art of cutting” using scalpels, scissors, and various other instruments and it poses risk of unintended injuries and infection to the surgeon and operating team as well as to the patient. Orthopedic surgery is not an exception of such risks and the risk has been calculated to be even higher than in many other branches of surgeries.

Forty years ago implantation of a total hip prosthesis (THA) was marked by a seven-percent infection rate at 6 months [3, 4]. Sir John Charnley understood at the time that reducing infections required improving practices in the operating room where contamination took place. Surgical asepsis, the use of laminar flow, and antibiotic prophylaxis, as well as cutaneous perforations, have greatly reduced intraoperative bacterial contamination [4], thus reducing the postoperative infection rate currently around 1% [5, 6]. Surgical gloving is the showpiece of this asepsis having the effect of protecting the surgical team from the patient's biological fluids and use of double glove is a recommended practice [1, 5–7].

Needle stick injuries are the most common source of blood contamination during surgery [8]. There is no vaccination available until now to protect against human immunodeficiency virus (HIV). Though vaccination against hepatitis B virus is now available, only 60% of surgeons are reported to be immunized against it [9, 10]. Besides this, transmission of hepatitis C has also been reported and many other diseases may be transmitted with biological fluids in the future. Keeping this in view a new “pointless” suture needle was developed. This needle has a blunt tapering point which allows tissue penetration with minimum force but does not puncture glove or skin.

Contamination of surgeon's hand from patient undergoing surgery is a potential source of occupationally acquired infection. Orthopedic and trauma surgeons are thought to be at particular risk [11, 12]. Latex gloves offer protection but are often punctured, rendering them ineffective [13–16]. There is evidence that wearing two pairs of latex gloves (double gloving) improves protection, but there is still a high rate of perforation of the inner glove [12, 13, 16, 17]. Cut resistant glove liner was developed to curb the rate of inner glove perforations in double gloving system.

Surgical gloves were invented by the German surgeon von Mikulicz and the first rubber glove was presented by the German surgeon Paul Leopold Friedrich a couple of years ahead of Halstead. Initially surgical gloves were used to protect the surgical team from getting infected, but later it gained importance in protecting the patient as well against infection. One study revealed positive results in cultures obtained from the periphery of the perforation site in 10 percent of perforated surgical gloves [18]. The frequent use of penetrating devices such as wires, saws, or needles during orthopedic procedures increases the risk for transmission of bloodborne infections like HIV, hepatitis B, and hepatitis C. Moreover, whenever there is disruption of integrity of skin it poses additional risk of contamination through a perforated glove. Palmer and Rickett determined that skin integrity was disrupted prior to surgery in 13 percent of the surgical teams [18]. Though using surgical gloves protects the surgical team members against bloodborne diseases, prolonged operation duration in conjunction with a perforated glove and disrupted skin integrity increases the contamination risks even higher.

Using indicator surgical gloves may be protective for the surgical team, especially during surgical procedures in risky cases [19], although double gloving systems with indicator are no guarantee to detect perforation. Microperforations on these gloves are easily recognizable and can thus be changed whenever perforated [20]. In addition, strengthened gloves are also protective in specific surgeries using penetrating instruments such as Ilizarov surgery, hip arthroplasty, and other major orthopedic surgeries.

The advent of parenterally transmissible diseases such as HIV and hepatitis B and the risks of their transmission during surgeries has resulted in reappraisal of surgical techniques and instruments and the need of general immunization of surgical team. Surveys have repeatedly shown that the penetrations of gloves may occur as frequently as one in three procedures, with penetrating skin injuries having an incidence of approximately one in fifteen surgical procedures. It cannot be simply dismissed as unavoidable hazard of the trade because not only does it carry real risks of infection transmission but also there is the effect of psychological stress on staffs, particularly if such injuries involve “high risk” patients.

## 2. Materials and Method

### 2.1. Selection of Cases

A total of 19 primary hip replacement arthroplasties done in the First Affiliated Hospital of Jiamusi University, Orthopedics Department, Unit 3, between March 2013 and November 2013, were included in the study. The study was of single gloving system as it was the common practice in this hospital. There were a total of 191 gloves collected from 19 primary hip replacement arthroplasties with 15 being total hip replacement arthroplasty and four hemiarthroplasty.

In all the surgeries the surgical team wore a single pair of latex glove of the same brand. In 13 surgeries, the operating team consisted of the surgeon, 1st, 2nd, and 3rd assistant, and a nurse. In the remaining 6 surgeries, the 3rd assistant was not used. Six gloves were changed for perforation during surgery and seven gloves were changed due to excessive soakage or contamination.

### 2.2. Research Methods

At the end of surgery all the gloves were collected, washed gently with water, then visually examined, and also examined using water inflation technique. Perforation of the gloves was tested using the water test (European standard EN 445) [21]. Each glove was filled with one litre of water and the cuff twisted through 360° to increase the pressure and to test for leakage. Relevant data regarding patient's age, sex, starting and ending time of operation, and any visible perforations occurring during surgery were noted. A total of one hundred and ninety-one surgical gloves were evaluated. The location, number and timing of perforations, and duration of each operation were taken into consideration. It was a level 3 prospective study.

### 2.3. Statistical Analysis

SPSS version 17.0 software was used for analysis and processing data; measured data were expressed as mean ± standard deviation (*x* ± *s*). The chi-square test was used to analyze the data to determine whether there is a significant difference between the expected frequencies and the observed frequencies in various categories.

The null hypothesis states that there is no significant difference between the expected and observed frequencies. The alternative hypothesis states that the expected and observed frequencies are different. Level of significance is 5% or 0.050 and a *P* value greater than 0.050 shows no association between the variables. The chi-square value was determined using the formula:

(1) $$\begin{array}{c} \chi^{2}=\frac{{\left(O-E\right)}^{2}}{E}, \end{array}$$

where *O* is the observed frequency in each category, *E* is the expected frequency in the corresponding category, and *χ* ^2^ is the chi-square value.

## 3. Results

There were a total of 191 gloves collected from 19 primary hip replacement arthroplasties with 15 being total hip replacement arthroplasty and four hemiarthroplasty.

The mean age of patient undergoing total hip replacement arthroplasty was 73.5 ± 5.9 years and the mean operation duration was 118.3 ± 19.5 minutes. All the surgeons were right handed (Table 1).

**Figure 1:**
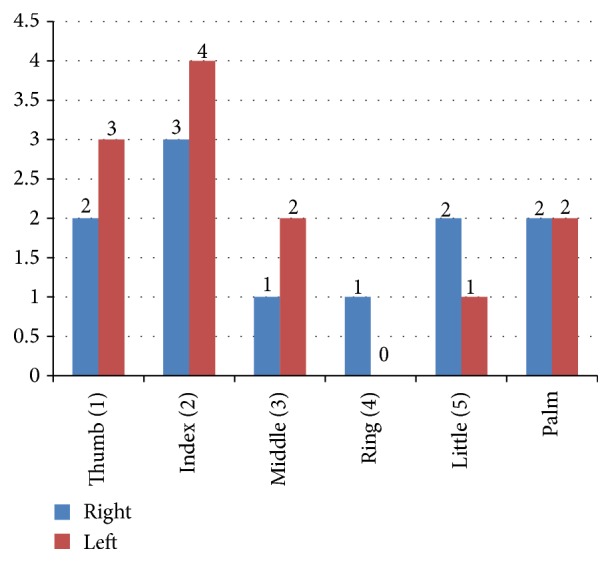
Histogram showing glove perforation by region of hand.

Glove perforation was detected in 23 gloves (12.04%) that had been utilized in 15 surgical procedures (78.9%) (Figure 1). In four of the primary total hip replacement arthroplasties no perforations in any of the gloves were detected.

**Figure 2:**
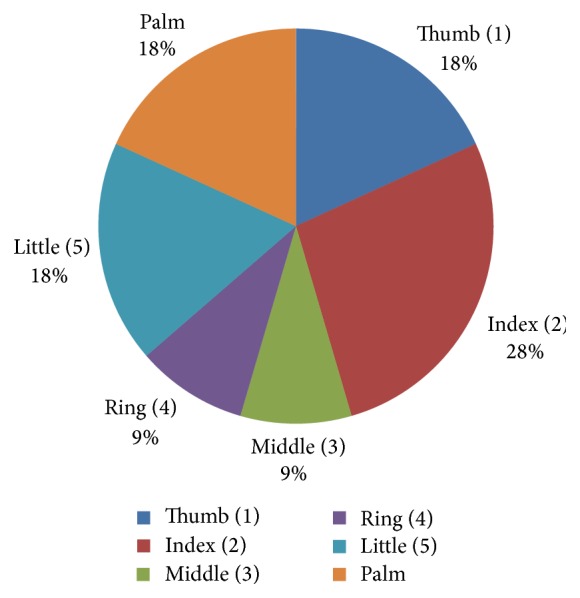
Pie chart illustrating percentage of glove perforation in different part of glove.

There were 12 glove perforations on left hand and 11 glove perforations on right hand. Glove perforations in index finger, thumb, and palm area accounted for 69.6% of total perforations (Figure 2).

During surgery six glove perforations were detected (26.1%). There was no significant difference between glove perforation in dominant hand and nondominant hand (with *P* < 0.05 being significant). However, surgeon and the first assistant incurred majority of the glove perforations (60.1%).

## 4. Discussion

In the literature, the frequency of glove perforations has been reported between 3.3% and 57% in elective orthopedic surgeries [7, 15, 22]. In orthopedics surgery the fractured sharp bony edges may be responsible for increased frequency of glove perforations as well as use of sharp instruments like electric saws, power drills, Kirschner wires, bone cutter, and so forth. The present study found a glove perforation rate of 12.04% placing the results of my study on the lower range. In this study of single glove technique gloves were changed whenever there was a visible perforation or glove was changed when excessively contaminated with surgical fluids. Studies have shown that wearing two pairs of surgical gloves can reduce the frequency of glove perforation and the rate of glove contamination significantly [23, 24] implicating that the results from the present study may have been better if double gloving was used.

The significance of detecting glove perforation lies not only in the fact that it helps to avoid cross transmission of infectious diseases like HIV, hepatitis B, hepatitis C, and so forth, but also in the fact that intraoperative infection to patient can lead to catastrophic and devastating effects by causing postoperative infection in patient. This is especially true in case of replacement arthroplasty such as hip. In our study, the detection rate of visible glove perforation was 26.1% meaning that those not detected were 73.9%. This finding complied with the results reported in the literature ranging from 58% to over 80% of perforations not being detected by the wearer [25–27]. Studies in the literature suggest that glove perforation risks increase during operations of over 90 minutes and that gloves should be preferably changed [18, 28]. Demircay et al. stated that the risk for glove perforation was higher in the second hour of arthroplasty surgery, especially during the closure stage, due to needle prick injuries [29]. In our study the mean time of detection of glove perforation was 80.3 ± 21.9 minutes, again complying with findings of earlier studies. However, the use of single gloving technique could have caused slightly earlier perforation than found in literature.

There is consensus in the literature that glove perforation occurs most commonly in the thumb and index finger of the nondominant hand. Such perforations have been attributed to the use of nondominant hand to directly hold the needle, the reduced bone, tissue, or extremity leaving the dominant hand to hold instruments that require fine motor coordination [6, 29]. Our present study found that 52.1% of gloves of nondominant hands were perforated and that glove perforations in index finger, thumb, and palm region accounted for 69.6% of total perforations (Table 2). However, in our study the difference between glove perforation between right and left hand was not significant (value of *P* < 0.05 being significant).

In this present study majority of the perforations were found in gloves worn by the surgeon and the first assistant (60.1%). This was, however, lower than that found by the study done by Ibrahim Kaya et al. (87.5%). There are other studies that have found higher rate of glove perforation in nurses as compared with operating staffs which differs from my findings.

Although using surgical gloves protects the surgical team against bloodborne diseases, such as hepatitis-B and HIV, prolonged operation duration in conjunction with a perforated glove increases the contamination risk [23, 24]. In our study the mean operation duration was 118.3 ± 19.5 minutes. Study done by Palmer and Rickett determined that skin integrity was disrupted prior to surgery in 13% of the surgeries which further increases the risk for contamination through the perforated gloves. In risky cases the use of indicator gloves may be protective as microperforations on these gloves are easily recognizable [20], even though double gloving systems with indicator are no guarantee to detect perforation. Moreover, studies have shown that strengthened gloves like those with liner have even better protective effects.

The limitation of our study was that it included only single gloving system. The study of contamination of the gloves was not done and the study was not randomized and blinded which could have introduced bias in the results.

## 5. Conclusion 

Glove perforation during surgery such as hip replacement arthroplasty is not an uncommon phenomenon. In this study of single gloving system glove perforation rate was 12.04% whereas literature reports of glove perforation rate as low as 3.3% in elective orthopedic surgeries with double gloving system. As such emphasis should be given to wear double pair of gloves wherever this practice is uncommon.

## Figures and Tables

**Table 1:** Data collected from 19 primary hip replacement arthroplasties.

Case number	Age and sex	Number and timing of visible glove perforation (detected during surgery) (*A*)	Number of nonvisible glove perforation (detected after surgery) (*B*)	Total perforated gloves (*A* + *B*)	Staffs with glove perforation
1	74F	1^*^ (83 min)	1^*^	2	Surgeon and 1st asst.
2	73M		1^*^	1	Nurse
3	82F		2	2	Surgeon and nurse
4	69F	1 (72 min)	0	1	1st asst.
5	83M		0	0	
6	68M		2^*^	2	Nurse and 2nd asst.
7	66F	1^*^ (64 min)	0	1	Surgeon
8	69M		0	0	
9	76M		1	1	Surgeon
10	78F		3	3	Nurse and 1st and 2nd asst.
11	81M	1^*^ (112 min)	1^*^	2	Surgeon and 2nd asst.
12	83F		1	1	3rd asst.
13	77M		2^*^	2	Surgeon and 1st asst.
14	73M	1 (98 min)	0	1	1st asst.
15	69F		2	2	Surgeon and 2nd asst.
16	72F	1^*^ (53 min)	0	1	Surgeon
17	71F		0	0	
18	68F		1^*^	1	1st asst.
19	64M		0	0	

Asterisk “∗” stands for glove perforation in left hand.

**Table 2:** Number of gloves perforated in different region.

	Thumb	Index finger	Middle finger	Ring finger	Little finger	Palm	Total
Right side	2	3	1	1	2	2	11
Left side	3	4	2	0	1	2	12

Total	5	7	3	1	3	4	23
